# Role of Plant-Specific N-Terminal Domain of Maize CK2β1 Subunit in CK2β Functions and Holoenzyme Regulation

**DOI:** 10.1371/journal.pone.0021909

**Published:** 2011-07-15

**Authors:** Marta Riera, Sami Irar, Isabel C. Vélez-Bermúdez, Lorenzo Carretero-Paulet, Victoria Lumbreras, Montserrat Pagès

**Affiliations:** 1 Department of Molecular Genetics, Centre for Research on Agricultural Genomics CRAG (CSIC-IRTA-UAB), Barcelona, Spain; 2 Department of Applied Biology (Area of Genetics). University of Almería, Spain; Tulane University Health Sciences Center, United States of America

## Abstract

Protein kinase CK2 is a highly pleiotropic Ser/Thr kinase ubiquituous in eukaryotic organisms. CK2 is organized as a heterotetrameric enzyme composed of two types of subunits: the catalytic (CK2α) and the regulatory (CK2β). The CK2β subunits enhance the stability, activity and specificity of the holoenzyme, but they can also perform functions independently of the CK2 tetramer. CK2β regulatory subunits in plants differ from their animal or yeast counterparts, since they present an additional specific N-terminal extension of about 90 aminoacids that shares no homology with any previously characterized functional domain. Sequence analysis of the N-terminal domain of land plant CK2β subunit sequences reveals its arrangement through short, conserved motifs, some of them including CK2 autophosphorylation sites. By using maize CK2β1 and a deleted version (ΔNCK2β1) lacking the N-terminal domain, we have demonstrated that CK2β1 is autophosphorylated within the N-terminal domain. Moreover, the holoenzyme composed with CK2α1/ΔNCK2β1 is able to phosphorylate different substrates more efficiently than CK2α1/CK2β1 or CK2α alone. Transient overexpression of CK2β1 and ΔNCK2β1 fused to GFP in different plant systems show that the presence of N-terminal domain enhances aggregation in nuclear speckles and stabilizes the protein against proteasome degradation. Finally, bimolecular fluorescence complementation (BiFC) assays show the nuclear and cytoplasmic location of the plant CK2 holoenzyme, in contrast to the individual CK2α/β subunits mainly observed in the nucleus. All together, our results support the hypothesis that the plant-specific N-terminal domain of CK2β subunits is involved in the down-regulation of the CK2 holoenzyme activity and in the stabilization of CK2β1 protein. In summary, the whole amount of data shown in this work suggests that this domain was acquired by plants for regulatory purposes.

## Introduction

Protein kinase CK2 is a constitutively active, highly conserved serine/threonine protein kinase that is ubiquitously distributed in eukaryotes. CK2 is one of the most pleiotropic kinases known, able to phosphorylate and interact with multiple cellular proteins [1], [2]. In mammals the typical CK2 holoenzyme is a heterotetrameric complex composed of two catalytic (CK2α and CK2α′) and two regulatory (CK2β) subunits. The CK2β regulatory subunits are inactive and present no homology to regulatory subunits or domains of other protein kinases. In the classical model of CK2 tetrameric holoenzyme, CK2β regulatory subunits are involved in the assembly of CK2 tetrameric complexes, in enhancing catalytic activity and stability of CK2α and in modulation of the substrate specificity of CK2 [3]. However, CK2β subunits also have additional functions in addition to regulation of the holoenzyme, since they can interact with and regulate other proteins in the absence of CK2α subunits [4], [5]. Structural analysis by X-ray crystallographic assays shows that CK2 tetramers are subject to disassembly and re-assembly [6]. In addition, localization studies of individual CK2 subunits indicate that both types of subunits have been found in different compartments [7], [8]. These findings indicate that individual CK2 subunits may have an independent role. All these evidences support the idea of the independent role of the individual CK2 subunits versus the classical holoenzyme.

In plants CK2 is involved in relevant processes such as plant growth and light-regulated gene expression [9], circadian rhythm [10], [11], cell-cycle regulation and development [12], [13], salicylic acid mediated defense [14] and abiotic stress responses [15]. CK2α/β subunits family is expanded in plant genomes relative to animal genomes, since they belong to multigenic families composed by up to 4 genes. As reported in animals, differential subcellular localization of plant CK2 subunits suggests specific functions for each CK2 subunit or CK2 isoform [15], [16]. This hypothesis is also supported by new findings showing that specific CK2 holoenzyme isoforms can regulate the initiation of translation in *Arabidopsis* [17]. In maize, three genes for each CK2α/β have been described to date [18]–[20]. A fourth CK2β gene (CK2β4) has been found in the Maize Genomic Database (MaizeGD) and is included in this paper. Since it was crystallized [21], maize CK2α1 subunit has been widely studied as a model of CK2 structure and it has been used successfully to design inhibitors of the holoenzyme [22]. This is due to the biochemical characteristics of maize CK2α, which is highly stable and has more specific activity than the human holoenzyme. Comparative studies demonstrate that the maize holoenzyme is less stable than the human counterpart [23]. However, despite copious data on CK2α, little is known about CK2β regulatory subunits and CK2 holoenzyme in maize. Plants have a greater diversity of CK2β subunits than animals or yeasts [24]. Although plant CK2βs preserve in their central core the characteristic CK2β features, they lacked 20 aminoacids from the C-terminal domain and contain a specific N-terminal extension of about 90 aminoacids. This N-terminal region shares no homology with any previously characterized functional domain. The absence of functional data about this domain prompted us to investigate its putative role in: (i) CK2β functions and (ii) CK2 holoenzyme regulation. Using maize CK2β1 and a deleted version lacking N-terminal domain (ΔNCK2β1) we demonstrate that this plant-specific N-terminal extension affects both CK2β and CK2 holoenzyme properties. In addition, we postulate a new role for CK2β subunits in plants, since CK2β1 releases CK2α1 subunits from the nucleolus and the CK2 holoenzyme can be found all over the cell. These findings show that *in vivo* localization of the plant CK2 holoenzyme is different from that of the independent CK2α/β subunits alone. Even though the N-terminal domain of CK2β is not involved in this export mechanism, the data reported here indicates a role of this domain in regulation of both CK2β subunits and CK2 holoenzyme in plants.

## Results

### Sequence and evolutionary analysis of the N-terminal domain of plant CK2β subunits

**Figure 1:**
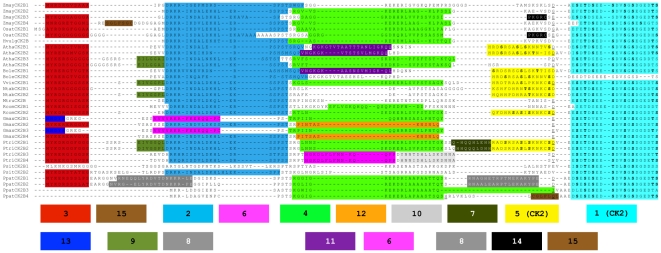
Multiple Sequence Alignment of N-terminal domains of land plant CK2β regulatory subunits. Conserved not-overlapping motifs identified in the MEME analysis are background-coloured. Positions in bold correspond to Serine and Threonine (S, T) residues predicted as CK2 phosphorylation sites. Location of the first intron is underlined.

All plant CK2β subunits display an extra domain located N-terminal to the highly conserved CK2β central region. These N-terminal CK2β domains are poorly conserved both in length and in primary sequence. At the amino acid composition level, they are significantly enriched in phosphorylable residues such as Ser (averaging ca. 10%), Thr and Tyr. Using the N-terminal domain of maize CK2β1 as a query, BLAST searches were performed in different protein databases, including the whole proteome of selected plant species (Table S1). As a result, 34 sequences corresponding to CK2β from 13 species representative of the main land plant evolutionary lineages were identified (Table S2). Additional searches of the protein databases were performed through HMMer using as a query a hidden Markov models (HMM) profile constructed on the basis of the alignment of 33 N-terminal domains. Despite HMM profiles perform better in detecting remote homologies [25], only land-plant species CK2β sequences were detected. The architecture of conserved motifs throughout the N-terminal domains was examined and represented over the corresponding alignment (Figure 1). Despite the high degree of divergence within the N-terminal domain, 15 short conserved motifs were found, some of them matching the consensus phosphorylation sites for specific protein kinases (Table S4), including putative CK2 autophosphorylation sites (motifs 1 and 5). Some motifs were highly conserved across almost every sequence examined (e.g. motif 1, particularly rich in acidic amino acids and including at least six Ser and/or Thr residues consensus of CK2 phosphorylation) while many others were apparently specific to certain evolutionary lineages (e.g. motif 5).

Genomic structure provides an independent criterion to assess the evolutionary relatedness among genes and functional domains. Exon/intron organization of plant CK2β for which the genomic sequences were available was determined. In all land-plant CK2β genes, location of the first intron was conserved at the same relative position of the N-terminal domain, just before motif 1. The first intron always showed phase 0 at the junction with exon 1 and phase 1 at the junction with exon 2 (Figure 1), supporting the acquisition of the N-terminal domain by land plants as encoded by a single exon.

To gain further insights into the evolutionary history of the land plant CK2β N-terminal domain, we performed a phylogenetic analysis of CK2β proteins from different eukaryotic kingdoms. For this purpose, we constructed a sequence dataset of 69 CK2β protein sequences, including sequences from animals and from several non-land plant species (algae, fungi, and protists) also displaying N-terminal extensions (Tables S2 and S3). Phylogenetic analyses were conducted using two independent methods: Neighbor Joining (NJ) and Maximum Likelihood (ML) [26]–[29]. A clade clustering all land-plant CK2β subunits could be unambiguously retrieved in both NJ and ML trees (Figure S1 and S2) and is clearly separated from other clades grouping CK2β from other organisms and also containing N-terminal extensions.

### The N-terminal domain of maize CK2β1 affects the CK2 holoenzyme activity

**Figure 2:**
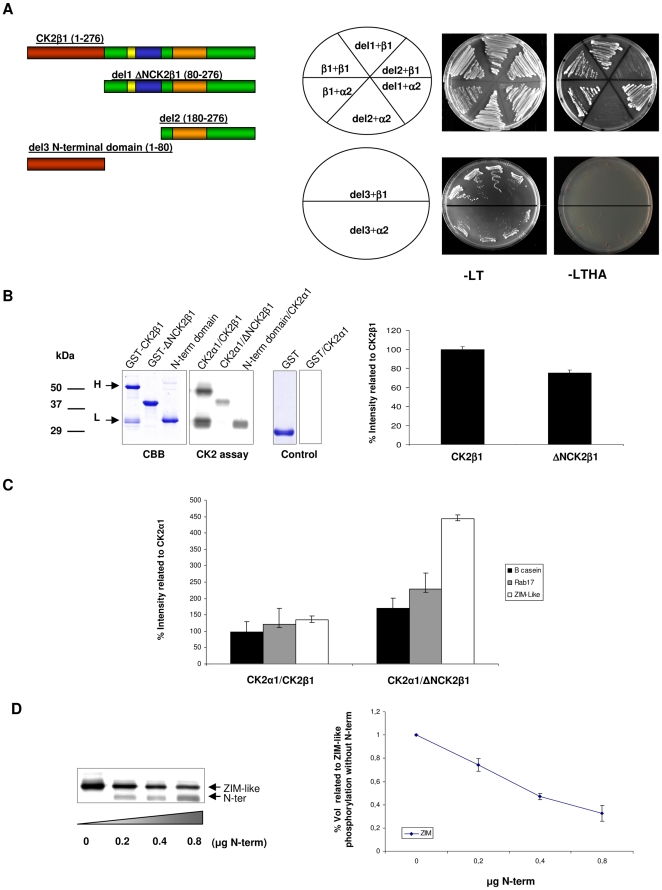
Intra-holoenzyme interactions using yeast two-hybrid system and in vitro CK2 phosphorylation assays using CK2 holoenzymes reconstituted with CK2α1 and regulatory subunits CK2β1 and ΔNCK2β1. (A) Left, Schematic representation of truncated versions of maize CK2β1 regulatory subunit used in the assay. Deletion 1, del1 ΔNCK2β1 (80–276): CK2β1 without N-terminal region, deletion 2 (del2) (180–276): CK2β1 without N-terminal region and acidic region and deletion 3 (del3) (1–80): N-terminal region alone. Right, Interactions between truncated versions of CK2β1 subunit and CK2α2/CK2β1 subunits with the two-hybrid system. The indicated transformants were selected in Leu-Trp plates and replated in selective plates lacking Leu-Trp-His-Ade. (B) Left panel, Gel stained with Coomassie Brillant Blue (CBB) containing the fusion proteins GST-CK2β1, GST-ΔNCK2β1 and GST-N-terminal domain (1–80) used in the autophosphorylation and CK2 phosphorylation assays. Middle panel, Autophosphorylation of reconstituted holoenzymes CK2α1/CK2β1, CK2α1/ΔNCK2β1 and in vitro phosphorylation of N-terminal domain (1–80) protein by CK2α1. In the first lane H, high molecular weight protein corresponding to fusion protein GST-CK2β1 (56 kDa) and L, low molecular weight protein, corresponding to intermediate products of about 30 kDa from fusion protein GST-CK2β1. Right panel, Coomassie Brillant Blue (CBB) and CK2 phosphorylation of GST protein alone (control). Right, Quantification of phosphorylated bands corresponding to CK2α1/CK2β1 and CK2α1/ΔNCK2β1 holoenzymes. 100% intensity corresponds to autophosphorylation of CK2β1. The data shown are calculated average values ± SD of three independent experiments. (C) Quantification of in vitro phosphorylation of β-casein, Rab17 protein and ZIM-like transcription factor by CK2α1/CK2β1 and CK2α1/ΔNCK2β1 reconstituted holoenzymes. 100% intensity corresponds to the phosphorylation of each protein by CK2α1 alone. The data shown are calculated average values ± SD of three independent experiments. (D), Left, In vitro phosphorylation of ZIM-like protein with CK2α1/ΔNCK2β1 (lane 1). In lanes 2 to 4 increasing amounts of CK2β1 N-terminal domain (1–80) has been added: 0.2 µg (lane 2), 0.4 µg (lane 3) and 0.8 µg (lane 4). Right, Relative phosphorylation of ZIM-like with the holoenzyme composed by CK2α1/ΔNCK2β1 with increasing amounts of CK2α1 N-terminal domain (1–80) (lanes 2–4) compared to phosphorylation of ZIM-like with CK2α1/ΔNCK2β1 holoenzyme (lane 1, assigned a value of 1). The data plotted (mean ±SD) represent three independent experiments.

To ascertain whether the plant specific N-terminal domain of maize CK2β1 affects CK2 holoenzyme regulation, we first analyzed if the domain is needed for CK2 holoenzyme assembly, CK2β/CK2β dimerization or interaction with CK2 substrates. We prepared constructs harbouring different deletions of the CK2β1 protein (Figure 2A) to perform two-hybrid assays. No significant interaction was detected between empty AD/BD-clone combinations (data not shown). Deletion del1 ΔNCK2β1 (80–276) strongly interacts with other CK2α catalytic subunits (CK2α2) as well as with full-length CK2β1. However, deletions del2 (180–276), corresponding to CK2β without N-terminal domain and acidic region and del3 (1–80), which corresponds to the N-terminal domain alone, do not interact neither with CK2α2 nor with CK2β1 subunits (Figure 2A). Therefore, these results demonstrate that CK2β N-terminal domain is not essential for intra-holoenzyme interactions.

We have previously demonstrated that recombinant maize CK2α and CK2β subunits can assemble in a functional tetrameric complex, and autophosphorylation of CK2β subunits demonstrates the functionality of the holoenzyme [20]. As previously observed in animals, dimerization of CK2β subunits seems to be a pre-requisite for holoenzyme formation [30]. Here we have reconstituted the active holoenzyme using the CK2α1 catalytic subunit and the deleted version of CK2β1 subunit (del1 ΔNCK2β1 (80–276)) and we found that in absence of the N-terminal domain of CK2β1, the CK2α1/ΔNCK2β1 holoenzyme is also functional and autophosphorylable (Figure 2B). Comparative CK2 autophosphorylation assays using holoenzymes CK2α1/CK2β1 and CK2α1/ΔNCK2β1 have been done and quantification of the autophosphorylation of both holoenzymes shows that CK2α1/CK2β1 was about 25% more phosphorylated than CK2α1/ΔNCK2β1 (Figure 2B, right). It is noteworthy that when GST-CK2β1 is overexpressed in *E coli*, a lower band (L) of about 30 kDa appears in addition to a higher band (H) corresponding to the fusion protein (56 kDa). Both bands are highly phosphorylated by CK2α *in vitro*. Purification and subsequent protein sequencing of this lower band demonstrate that it corresponds to intermediate products containing the N-terminal region of CK2β1. Moreover, the region corresponding to the CK2β1 N-terminal alone (1–80) fused to GST (fusion protein of 31 kDa) and overexpressed in *E coli* was also highly phosphorylated by CK2α1 *in vitro* (Figure 2B). Taken together, all these results suggest that autophosphorylation of CK2β1 occurs in high proportion at the residues located in the N-terminal domain.

To test whether CK2 activity was affected by the N-terminal domain of CK2β subunits, we compared the ability of both CK2 holoenzymes (CK2α1/CK2β1 and CK2α1/ΔNCK2β1) to phosphorylate *in vitro* substrates as β-casein, *in vivo* substrates as Rab17 or interacting partners as maize transcription factor ZIM-like. Interestingly, the holoenzyme composed by CK2α1/ΔNCK2β1 is able to phosphorylate β-casein, Rab17 and ZIM-like in greater amount than CK2α1 alone or CK2α1/CK2β1 (Figure 2C). These results points towards a possible role of the N-terminal domain of CK2β subunits as a negative regulator of CK2 activity. To confirm this hypothesis, we added increasing amounts of CK2β1 N-terminal domain (1–80) to the *in vitro* phosphorylation assays with the holoenzyme composed by CK2α1/ΔNCK2β1. The addition of exogenous CK2β1 N-terminal domain to the CK2α1/ΔNCK2β1 holoenzyme decreases its phosphorylation efficiency towards the substrates tested, ZIM-like (Figure 2D), β-casein and Rab17 (Figure S3). In conclusion, these results suggest that the N-terminal domain of CK2β subunits competes with the substrate for phosphorylation and down-regulate CK2α activity.

### The N-terminal domain of CK2β1 enhances stability of CK2β1 against proteasome degradation

**Figure 3:**
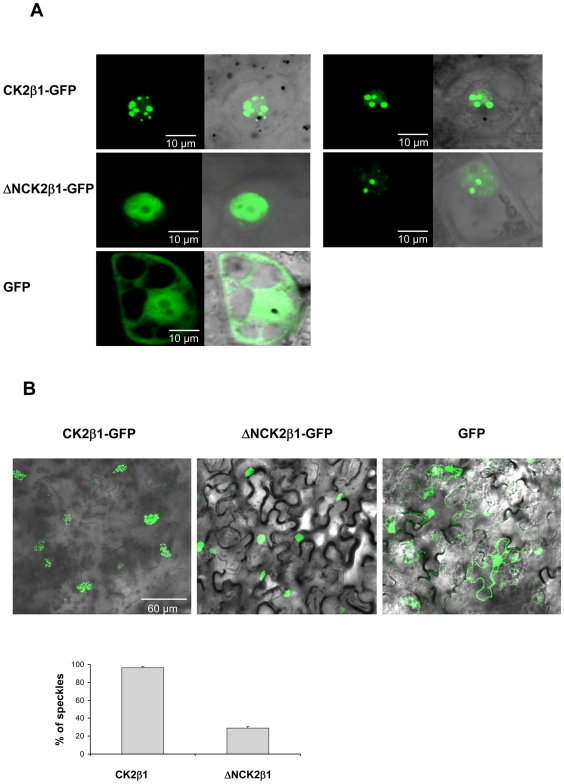
Subcellular localization of CK2β1-GFP and ΔNCK2β1-GFP in maize immature embryos (10 DAP) and Agrobacterium-infiltrated tobacco leaves. (A) Epifluorescence and bright-field images (merged with epiflourescence) (60×) of 10 DAP embryos cells transformed by particle bombardment with the indicated constructs (CK2β1–GFP, ΔNCK2β1-GFP and GFP alone). (B) Upper, General views (40×) of Nicotiana benthamiana leaves infiltrated with a mixture of Agrobacterium suspensions harbouring the indicated constructs (CK2β1–GFP, ΔNCK2β1-GFP and GFP alone) and the gene silencing suppressor HcPro. Bottom, Quantification of cells presenting speckled pattern in cells transformed with CK2β1 and ΔNCK2β1. The graphic representation correspond to average for data corresponding to 3 independent experiments ±SD (n = 100).

To determine whether the N-terminal domain of CK2β subunits is involved in regulation of their subcellular localization, the deleted version of CK2β1 (del1 ΔNCK2β1 (80–276)) was fused to GFP and examined by confocal microscopy in different plant systems: immature maize embryos (10 DAP) transformed by particle bombardment, agroinfiltrated tobacco leaves and onion epidermal cells (Figure 3 and Figure S4).

In all plant systems examined the results obtained show that both CK2β1 and ΔNCK2β1 are mainly located in the nucleus, but whereas in the transformation with CK2β1 most of cells presented nuclear speckles, in cells transformed with ΔNCK2β1 we found two different patterns: cells presenting a diffuse nuclear pattern as well as cells showing nuclear speckles. Any nuclear speckle structures were observed in cells transformed with GFP alone. Since the total number of transformed cells after maize bombardment is much lower than in tobacco cells, we counted the percentage of ΔNCK2β1 cells presenting speckles *vs.* a diffuse pattern in agroinfiltrated tobacco leaves (Figure 3B). Only 29% of cells transformed with ΔNCK2β1 presented speckles *vs.* 71% with diffuse pattern, whereas for cells transformed with full-lenght CK2β1 96% of the cells presented speckles. Therefore, the absence of nuclear aggregates in the ΔNCK2β1 cells could be linked to the deletion of the N-terminal domain.

**Figure 4:**
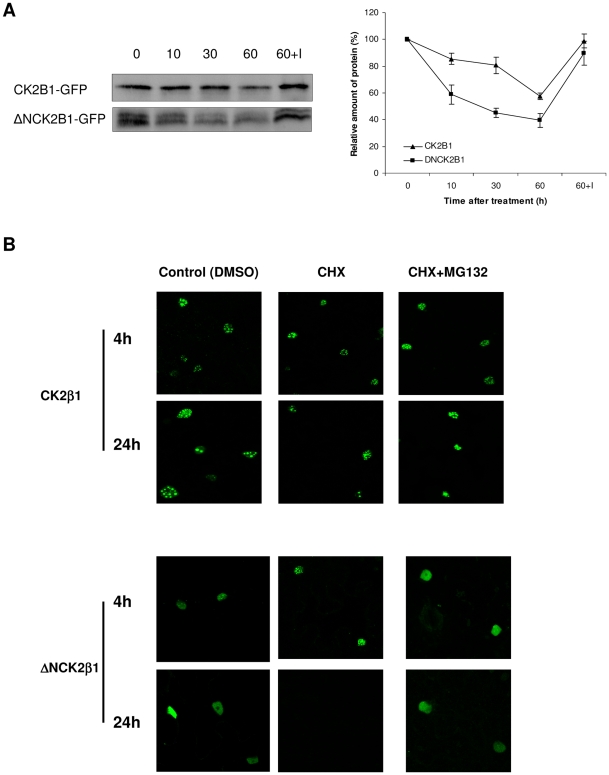
Protein degradation of CK2β1 and ΔNCK2β1. (A) Immunodetection of CK2β1-GFP protein and ΔNCK2β1-GFP protein in transformed N. benthamiana leaves using anti-GFP antibody. Protein extracts were incubated at 30°C in an in vitro degradation buffer (see Experimental procedures) with or without proteasome inhibitor (MG132) for the indicated time (min). 30 µg of total protein was loaded onto gels. 60+I indicates extracts treated with 100 µM MG132. Each signal strength was measured by Quantity One and plotted in the right panel as the relative amount of remaining protein. Quantitative data (mean ±SD) represent three independent experiments. (B) General views (40×) of Nicotiana benthamiana leaves infiltrated with CK2β1-GFP and ΔNCK2β1-GFP with different treatments: control, cycloheximide treatment (CHX, 50 µM), and CHX (50 µM)+ proteasome inhibitor MG132 (100 µM) combination treatment after 4 and 24 hours. The images shown are representative of more than 5 independent experiments.

To test if the better ability of CK2β1 *vs.* ΔNCK2β1 to form nuclear aggregates affects the protein stability, we performed cell-free degradation assays. Total protein extracts from tobacco leaves transformed with CK2β1 and ΔNCK2β1 fused to GFP were maintained for 10, 30, 60 min at 30°C without protein inhibitors, and aliquots were analyzed by Western blot using anti-GFP antibody (Figure 4A, left). Results obtained suggest that the amount of both CK2β1 and ΔNCK2β1 decreased over time. Subsequently, we added proteasome inhibitor MG132 to test whether the degradation observed was due to the proteasome pathway. In samples treated with MG132 the fusion protein remained stable, indicating that MG132 protects CK2β1 and ΔNCK2β1 against proteasome degradation. The relative amount of remaining proteins was estimated from these data and plotted, and the rates of protein degradation for ΔNCK2β1 was considerably higher than the rates for CK2β1 (Figure 4A, right). These results suggest that the protein lacking the N-terminal domain is more susceptible to degradation by proteasome than the full-length CK2β1. To examine the effect of the N-terminal domain on CK2β1 degradation by the proteasome pathway, we treated transformed tobacco leaves with cycloheximide (CHX) to inhibit *de novo* protein synthesis and we observed samples by confocal microscopy for up to 24 h (Figure 4B). After 4 h of treatment with CHX, the immunofluorescent signal was visible in both CK2β1 and ΔNCK2β1 samples. In parallel, we have taken samples of treated cells at different times and analyzed them by Western blot. In agreement with the results obtained by confocal analysis, the *in vivo* stability at short times is similar for both proteins (Figure S5). However, after 24 h, we detected the immunofluorescent signal only in cells transformed with CK2β1, indicating the requirement of ongoing protein synthesis to maintain steady-state levels of ΔNCK2β1 protein. When samples were treated with MG132 and CHX+MG132, we are able to detect cells transformed with ΔNCK2β1 after 24 h of treatment, indicating that proteasome inhibition protects ΔNCK2β1 from degradation.

### Localization of CK2α1/CK2β1 holoenzyme is different from its CK2 individual subunits

**Figure 5:**
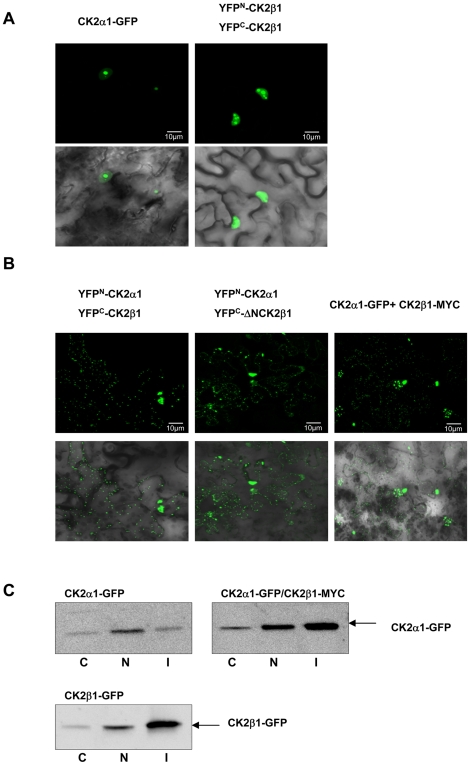
Subcellular localization of CK2 individual subunits and CK2 holoenzyme in leaf epidemis of N. benthamiana plants. (A) General views (40×) of Nicotiana benthamiana leaves co-infiltrated with a mixture of Agrobacterium suspensions harbouring the indicated constructs: CK2α1-GFP (left), YFP^N^-CK2β1/YFP^C^-CK2β1 (right) together with gene silencing suppressor HcPro. (B) General views (40×) of Nicotiana benthamiana leaves co-infiltrated with Agrobacterium containing the gene silencing suppressor HcPro and the following pair constructs: YFP^N^-CK2α1/YFP^C^-CK2β1 (left), YFP^N^-CK2α1/YFP^C^-ΔNCK2β1 (middle), and CK2α1-GFP/CK2β1-Myc (right). In panel A and B upper correspond to epifluorescence images and bottom to bright-field images (merged with epiflourescence). (C) Immunodetection of CK2α1-GFP and CK2β1-GFP proteins using anti-GFP antibody in N. benthamiana leaves transformed with CK2α1-GFP, CK2α1-GFP/CK2β1-Myc and CK2β1-GFP. C corresponds to cytosolic fraction, N to nuclear fraction and I to insoluble fraction (including nuclear and cytosolic aggregates).

Different localization of plant CK2 subunits have been previously demonstrated [16]. In maize all CK2α subunits described to date (CK2α1 to CK2α3) present nuclear localization with high accumulation in nucleolus; whereas CK2β1 and CK2β2 are mainly located in nuclear speckles and CK2β3 can be found in both nucleus and cytoplasm [15]. However, nothing is known about plant CK2 holoenzyme localization. To investigate that, we conducted Bimolecular Fluorescence Complementation (BiFC) assays in agroinfiltrated tobacco leaves [31], [32]. In that system, CK2α1 and CK2β1 split YFP tagged proteins must interact *in vivo* to reconstitute YFP fluorescence. In CK2 heterotetramer the two CK2β subunits associate as a stable dimer in the core of the holoenzyme whereas the two CK2α are located in the external part without interacting among themselves [29]. Using BiFC we show that CK2β1 subunits dimerize and present the same nuclear speckled localization described for CK2β1 fused to GFP (Figure 5A). Interestingly, the CK2 holoenzyme CK2α1/CK2β1 is located not only in nucleus but also in cytoplasmic aggregates (Figure 5B). Next, we performed BiFC reconstituting the holoenzyme with CK2α1 and ΔNCK2β1. As expected, ΔNCK2β1 interacted with split CK2α1, being also found in nucleus and cytoplasm, as in the case of CK2α1/CK2β1 holoenzyme (Figure 5B). To confirm the presence of the CK2 holoenzyme in the cytoplasm, we perform an alternative approach by co-transfecting tobacco leaves with CK2α1-GFP and CK2β1 fused to a non-fluorescence tag (Myc). This method allows to verify that the fluorescence signal detected in the cytoplasm is due to the presence of CK2α1-GFP in this compartment. Since CK2α1-GFP/CK2β1-Myc holoenzyme is also located in nucleus and cytoplasm, we confirmed that CK2β1-Myc is able to modify CK2α1-GFP localization from nucleus/nucleolus to nucleus and cytoplasm aggregates. In addition, we have used plants co-transfected with CK2α1-GFP/CK2β1-Myc to perform subcellular fractionation and Western blot analysis using anti-GFP antibody (Figure 5C). In control plants overexpressing CK2α1-GFP alone, CK2α1 subunit was mainly detected in nuclear soluble fraction (N) whereas in plants co-transfected with CK2α1-GFP/CK2β1-Myc, CK2α1 is increased in the insoluble fraction (I), which includes all insoluble particles from nucleus and cytoplasm. These results suggest that CK2β1-Myc is able to shift CK2α1-GFP from nuclear fraction to insoluble aggregates in both nucleus and cytoplasm.

## Discussion

Land plant CK2β subunits show distinctive features from their eukaryotic counterparts, including the formation of expanded families, shorter C-terminal domains and longer N-terminal domains. Preliminary *in-silico* analysis of the plant specific N-terminal domain indicates that it presents no homology with other protein or domains either in sequence or in structure. In addition, prediction programs were unable to determine a secondary structure for this domain. In an attempt to understand the role and functionality of this domain in plants, we performed a complete sequence analysis. The domain is arranged through short, conserved motifs, many of them putatively corresponding to specific kinase phosphorylation sites, including CK2 autophosphorylation sites. The domain may have evolved through the gain and loss of short conserved motifs, resulting in a mosaic pattern. The occurrence of N-terminal extensions is not exclusive of land plants, having been also found in fungi, intracellular protozoan parasites and algae. However, we did not found any protein or domain outside land plant CK2βs showing significant identity at the sequence level with the N-terminal domain. Moreover, phylogenetic analysis shows a separated clade clustering all land plant CK2β subunits. As expected, green and red algae CK2β sequences clustered at the base of the land plant clade. However, branching of the single representative from the red algae *Cyanidioschyzon merolae*, which diverged from other photosynthetic eukaryotes 1.5 billion years ago, was less bootstrap supported [33]. Also protists and fungi are separated from land plants in both phylogenetic trees, in accordance to previously reported data that demonstrate the early diverging evolution of CK2β from plants [34]. Furthermore, the exon/intron structure of genomic sequences encoding for the CK2β N-terminal domain was absolutely conserved in all land plant CK2β genes analyzed. All together, these results support the independent acquisition of the N-terminal domain by land plants as a single exon. Further evolutionary diversification of land plant CK2β would have involved differential gene family expansion, which may have promoted the acquisition of additional functional specificities by multiplying the number of putative regulatory networks in which they could be involved.

Despite of the elucidation of maize CK2α catalytic structure, no structure for the plant CK2β regulatory subunit has been reported to date. Here, by using a two-hybrid approach we show that the CK2β N-terminal domain did not affect intra-molecular (CK2α/β or CK2β/β) holoenzyme interactions. These results indicate that the N-terminal domain is located in the external part of the holoenzyme, although structural studies such as the crystallization of the CK2β regulatory subunit would be needed to localize it with greater precision.

Most animal CK2β subunits are autophosphorylated only at two highly conserved residues, Ser^2^ and Ser^3^ [35]. This consensus is only partially conserved in plants: in all plant species Ser^2^ is conserved as Ser residue (Ser^83^ in maize CK2β1), but Ser^3^ is replaced in all plant sequences analyzed by acidic residues (Asp or Glu). In contrast to animals, land plant CK2β subunits present additional putative autophosphorylation sites (motifs 1 and 5). For instance, maize CK2β1 has five additional Ser residues at the motif 1 of the N-terminal domain that might be targets for CK2 autophosphorylation. Motif 1 is rich in Asp and Glu residues and is one of the most conserved in all plant N-terminal sequences (Figure 1). In addition, CK2β1 subunits present additional Ser residues located in the central core of the protein not present in animal CK2β proteins. Our *in vitro* phosphorylation assays show that the holoenzyme reconstituted with CK2α1 and CK2β1 is higher autophosphorylated than the holoenzyme with CK2α1 and ΔNCK2β1 (Figure 2B). Moreover, the N-terminal domain alone is highly phosphorylated by CK2α1 *in vitro*. Taken together, these results suggests that the putative CK2 consensus sites located in the N-terminal domain are functional and might be involved in regulating CK2 activity. *In vitro* phosphorylation assays showed that when the holoenzyme is reconstituted with CK2α1 and ΔNCK2β1 the phosphorylation of several substrates is enhanced. These results point towards a possible role of the N-terminal domain of CK2β down-regulating CK2α subunit activity. The competition assays using the N-terminal fragment support this hypothesis. The N-terminal extension of the protist *Plasmodium falciparum* has also been postulated to act as a down-regulator of CK2α subunits [36], even though our analysis supports the independent origin of the N-terminal domain of land plant CK2β.

The greater efficiency of the maize holoenzyme without the N-terminal domain is also consistent with the results of our previous studies comparing human *vs.* maize holoenzyme, which demonstrated a high stability and high specific activity of human CK2 holoenzyme (without N-terminal domain) compared to its maize counterpart [23]. It has been recently reported that a splicing variant of maize CK2α1 (named CK2α-4) could act as a specific negative regulator of CK2 activity [37]. Taken together, all these results suggest that maize CK2 activity could be regulated by different mechanisms involving both CK2α/β subunits.

Functional studies were performed in order to assess whether the presence of the N-terminal domain has a role in regulation of CK2β subcellular localization. Our results show that maize CK2β1 is highly prone to aggregation in nuclear speckles and the deletion of N-terminal domain decreases this accumulation of CK2β1 in stable nuclear aggregates. It has been reported for other proteins such as mammalian PGC-1α and transcription factor ATF4 that aggregation in nuclear bodies protects against proteasome degradation [38], [39]. Here we show that maize CK2β1 is also degraded by the ubiquitin-dependent proteasome pathway as described for *Arabidopsis* CK2β4 [40]. Interestingly, cell-free degradation assays show that deletion of the N-terminal domain increases the rate of CK2β1 protein degradation. Our findings indicate a role for the N-terminal domain in enhancing CK2β1 aggregation in nuclear speckles, where the protein is assumed to be tightly complexed and less accessible to degradation machinery. Nevertheless, although the N-terminal domain can be considered as an “enhancer” of CK2β1 protein aggregation, it is not essential since ΔNCK2β1 can also aggregate. Thus, we can consider that aggregation in nuclear speckles protects CK2β1 against fast degradation by proteasome even though the protein is eventually degraded.

In human cells, CK2β is normally expressed at a higher level than CK2α catalytic subunits, allowing part of CK2β to be incorporated and stabilized into CK2 tetramers, whereas the excess CK2β is rapidly degraded with a half-life of less than 1 h [41]. Our results indicate that maize CK2β1 regulatory subunits are more stable than their animal counterparts probably due to their aggregation in nuclear speckles. Since the nature of these aggregates remains unclear, further experiments should be done to elucidate their composition and functional role.

We have previously demonstrated that different localization of the individual maize CK2α and CK2β isoforms [15] but nothing was known about holoenzyme localization in plants. Here by using BiFC we show the *in vivo* localization of CK2 holoenzyme in plant cells. Whereas individual subunits CK2α1 and CK2β1 present a nuclear localization, the holoenzyme CK2α1/CK2β1 is assembled in nucleus and is exported to the cytoplasm, where is complexed in aggregates. After analyzing the localization of the holoenzyme reconstituted with ΔNCK2β1, we conclude that the N-terminal domain is not involved in this export to the cytoplasm. In mammals it has recently been reported that CK2β regulatory subunits are required for the export of the holoenzyme as an ectokinase bound to the external surface of the cell membrane [42]. The same authors postulate a role of CK2β exporting not only CK2α but other CK2 interacting proteins. Our results implicate CK2β in the shift from nucleus/nucleolus to cytoplasm of CK2α subunits in plants. Further experiments may elucidate whether this export mechanism also involves other proteins.

In conclusion, our research shed new light on the regulation of protein kinase CK2 in plants. The whole amount of data shown in this work suggests that the plant-specific N-terminal domain of CK2β subunits was acquired in plants, as a single exon, for regulatory purposes, particularly in terms of regulation of holoenzyme activity and stabilization.

## Materials and Methods

### Plant CK2β regulatory subunits sequence analysis

Search for CK2β protein sequences was performed through BLAST and HMMER [43], [44]. Protein sequences were aligned using CLUSTALW and MUSCLE and the resulting alignments further edited through the MEGA 4.0 Alignment Explorer tool [45]–[47]. The MEME v. 3.5.7 tool was used to search for repeated sequence patterns (motifs) conserved across proteins [48]. Settings were changed to search for short motifs (3–20 aminoacids) showing any number of repetitions per sequence and position (p-values<1e-4). Search for functional domain and motifs was performed through the PROSITE and INTERPRO databases [49]. NetPhosK v1.0 server was used to predict kinase specific phosphorylation sites [50]. The location, distribution and phases of introns at the genomic sequences encoding for the N-terminal CK2β domain were determined using GENEWISE [50], [51]. Phylogenetic analyses performed are detailed in Text S1.

### Yeast two-hybrid assays

The Matchmaker two-hybrid system (Clontech) was used to perform yeast two-hybrid assays. For the two-hybrid assays, truncated versions of CK2β1 (del1 ΔNCK2β1 (80–276) del2 (180–276), and del3 N-terminal domain (1–80)) were generated by PCR and cloned into pGBT9 or pGBTK7 vectors into EcoRI/SalI sites. The specific primers used were detailed in Table S5. The other two-hybrid constructs used in the assays (pGBT9-CK2β1, pGAD424-CK2β1 and pGAD424-CK2α2) were previously described in [20]. Yeast (AH109 strain) transformation was performed according to the manufacturer's instructions. Yeast cells were cotransformed with the different pairs of BD-AD constructs and transformants were selected on minimal synthetic dropout medium (SD) -Leu-Trp (SD-LT). To test for protein-protein positive interaction, independent colonies were transferred to SD- -Leu-Trp-His-Ade (SD-LTHA).

### Recombinant protein expression and purification and *in vitro* autophosphorylation and CK2 activity assays

For expression and purification of recombinant CK2β proteins, the cDNAs of full-length CK2β1, del1 ΔNCK2β1 (80–276) and del3 N-terminal domain (1–80) were digested from pGBT9/pGBTK7 vectors using EcoRI/SalI sites and cloned in expression vector pGEX-4T-1 in frame to GST protein. The constructs were transformed into *E.coli* BL21(DE), and the proteins were expressed and purified as GST (Glutathione-S-Transferase) fusions as previously described [20] and according manufacturer's manual. Protein concentration of purified proteins (GST-CK2β1, GST-ΔNCK2β1 and GST-N-terminal domain) was determined by Bioanalyzer methods (Agilent technology) according to the manufacturer's instructions.

For the *in vitro* autophosphorylation assay, the holoenzymes CK2α1/CK2β1 and CK2α1/ΔNCK2β1 were reconstituted using 100 ng CK2α1 (Kinase Detect, Denmark) and 400 ng of GST-CK2β1 or GST-ΔNCK2β1 in a total volume of 30 µl CK2 buffer (8.9 mM MgCl_2_, 0.5 mM EGTA, 27 mM β-glycerol phosphate, 0.5 mM EDTA, 1 mM DTT, 0.08 mM ATP, 3 µCi of [γ-^33^P]ATP (3000 Ci/mmol). In the case of the CK2 activity assays, the holoenzymes CK2α1/CK2β1 and CK2α1/ΔNCK2β1 were reconstituted as described for the autophosphorylation assays and 0.6 µg of the different substrates tested (β-casein, GST-N-terminal domain, Rab17 or ZIM-like)were added to the reaction. In the competition assays, increasing amounts of N-terminal domain (1–80) (from 0.2 to 0.8 µg) were added to the reaction containing CK2α1/ΔNCK2β1 holoenzyme and 0.6 µg of β-casein, Rab17 or ZIM-like as substrates. In all cases, the samples were incubated for 30 min at 30°C. Reactions were stopped by addition of electrophoresis sample buffer, and the phosphorylated proteins were separated by 12% SDS–PAGE, visualized by PhosphoImager analysis and the intensity of the phosphorylated bands obtained was quantified by Quantity One (Bio-Rad) software according the manufacturer's suggestions.

### Cell-free degradation assays, western blot analysis and subcellular fractionation

The *in vitro* cell-free degradation assays was modified from [40]. 0.2 g transformed *N. benthamiana* leaves with CK2β1-GFP and ΔNCK2β1-GFP were ground in liquid nitrogen and resuspended in buffer A (50 mM Tris-HCl pH 7.5, 100 mM NaCl, 10 mM MgCl_2_, 5 mM DTT and 5 mM ATP). Equal amounts of extracts were transferred to individual tubes and incubated at 30°C and aliquots were taken at 20, 40 and 60 min. One aliquot was incubated with 100 µM of protein inhibitor MG132 (Enzo, Life Sciences, Inc.) for 1 h at 30°C. Reactions were stopped by adding protein gel-loading buffer. For Western blot analysis, proteins were electrophoresed on 12% SDS-PAGE gels, transferred to immobilon-P membranes (Millipore) and incubated with purchased antibodies against GFP (Invitrogen). The immunocomplexes were revealed using the ECL detection kit system (Super Signal West Femto, Pierce). Subcellular fractionation was done according to [38]. Briefly, transformed tobacco leaves were excised, ground in liquid nitrogen and resuspended in hypotonic buffer (10 mM HEPES, pH 7.9, 10 mM KCl, 0.1 mM EDTA, 0.1 mM EGTA, 1 µM dithiothreitol, and a protease inhibitor cocktail (1.6 mM aprotinin, 50 mM leupeptin, 1 mM pepstatin, 10 mM E-64 and 1 mM PMSF)). The extracts were homogenized and centrifuged at 10,000 rpm for 1 min. The supernatant was collected as the cytosolic fraction (C). The pellet was extracted in a high salt buffer (20 mM HEPES, pH 7.9, 400 mM NaCl, 1 mM EDTA, 1 mM EGTA, 1 µM dithiothreitol, and a protease inhibitor cocktail), and the soluble fraction was collected as nuclear extracts following another centrifugation (N). The remaining insoluble pellet was resuspended in SDS lysis buffer (I).

### Transient expression of GFP fusions in maize, *Nicotiana benthamiana* leaves and onion cells

For transient expression of GFP fusions in maize, tobacco and onion cells CK2β1 and del1 ΔNCK2β1 cDNAs were amplified by PCR using specific primers (Table S5) and cloned into binary vector pCAMBIA1302 under the control of a CamV 35S promoter and fused in the 3′ region with the GFP using BglII-SpeI sites for CK2β1 and BglII site for ΔNCK2β1. Additionally, the cDNA CK2β1 was amplified by PCR using specific primers (Table S5) and cloned in pLOLA vector [52] into BglII site in frame with Myc tag. The fusion CK2β1-Myc was transferred to pCAMBIA2300 using KpnI restriction site. For maize transformation immature maize embryos about 1 mm long were aseptically dissected from ears of field-grown maize plants (AxBxB73) after 10 days of pollination (10 DAP). Isolated embryos were placed o/n at 24°C in plates containing MS medium supplemented with 2.2 mg/L of 2,4D. 4 h before transformation embryos were moved to MS plates with 16 g/L of mannitol and were transiently transfected with GFP constructs by particle bombardment using the Biolistic PDS-1000/He Particle Delivery System (Bio-Rad). Plasmid DNA containing the different constructs was precipitated onto gold particles using CaCl_2_ and spermidine, and 1.5 µg DNA was delivered into intact maize tissue. After 24 h, the fluorescence of the bombarded cells were viewed using a FV 1000 confocal microscope (Olympus, http://www.olympus.com/).The same methodology was used to visualize the GFP fusion protein in epidermal onion cells. Young, fully expanded leaves from 5 week old tobacco plants were transiently transfected with *Agrobacterium tumefaciens* GV3101/pMP90 transformed with the GFP construct together with the silencing suppressor HcPro as has been described in [30], [31]. After 3–4 days, infiltrated areas from leaves were excised and examined by FV 1000 confocal microscopy (Olympus). For treatment with cycloheximide (CHX) and protease inhibitor MG132 leaves were excised and placed in sealed Petri dishes submerged into the solutions containing CHX 50 µM and MG132 100 µM in 2 ml of phosphate buffer. Treated and control samples were ground in liquid nitrogen and resuspended in buffer A (50 mM Tris-HCl pH 7.5, 100 mM NaCl, 10 mM MgCl_2_, 5 mM DTT, 5 mM ATP, and protease inhibitor cocktail) and analyzed by Western blot analysis as described above.

### Bimolecular fluorescence complementation (BiFC) assays

For BiFC assays, the cDNAs corresponding to the CK2α1, CK2β1 and ΔNCK2β1 were cloned in the GATEWAY-compatible vector pENTRY3C (Invitrogen). The cDNA CK21 was amplified by PCR using the specific primers detailed in Table S5 and the PCR fragment was cloned into BamHI-XhoI sites The cDNAs of full-length CK2β1, and del1 ΔNCK2β1 (80–276) were digested from pGBT9 vector using EcoRI/SalI sites and transferred to pENTRY3C. The three pENTRY3C plasmids were recombined by Gateway reaction into pYFP^N^43 and pYFP^C^43 vectors (kindly provided by A. Ferrando, University of Valencia, Spain, http://www.ibmcp.upv.es/FerrandoLabVectors.php.) to produce YFP^N^-CK2β1, YFP^C^-CK2β1, YFP^N^-CK2α1 and YFP^C^-ΔNCK2β1. Transformation of *N.benthamiana* leaves and visualization was performed as described above for transient expression of GFP fusions.

## Supporting Information

Text S1 **Phylogenetic analysis of CK2β regulatory subunits.** Phylogenetic analyses were performed on the basis of amino acid sequence alignments using two independent methods: Neighbor Joining (NJ) and Maximum Likelihood (ML). NJ analyses were implemented in MEGA 4.0 using the default settings [47] Prior to ML analysis; the best-fitting amino acid substitution model was selected using the Akaike information criterion as implemented in ProtTest v1.4 [26]. The resulting model: JTT with (i) an estimated proportion of invariable sites and (ii) a heterogeneous distribution of substitution rates across proteins with eight categories and an estimated shape parameter, was implemented in PHYML v3.0 to infer ML trees, using the subtree pruning and regrafting option to optimize tree topology searching [27]–[29]. To provide confidence on the resulting tree topology, a bootstrap analysis with 1,000 and 100 replicates in NJ and ML analyses, respectively, was performed.
(DOC)

Table S1 **Summary of genome databases searched for CK2β protein kinases.**
(DOC)

Table S2 **Summary of 34 land plant CK2β sequences.** Sequence identifier refers to the UNIPROT database, excepting for species examined independently, in which case the accession from the corresponding database was indicated (Table S1). The * designs sequence incomplete at its N-terminal end. Some genes have been identified to encode for alternatively spliced variants. In such cases, only a single representative protein sequence is shown.
(DOC)

Table S3 **Summary of 7 algae, 14 animal, 12 fungal and 2 protists CK2β sequences from representative species.** Sequence identifier refers to the UNIPROT database, excepting for species examined independently, in which case the accession from the corresponding database was used (Table S1). The * indicates sequences incomplete at its N-terminal end.
(DOC)

Table S4 **Summary of conserved motifs identified by MEME in plant CK2β subunits.** Matches of motifs with specific kinase phosphorylation sites, predicted by NetPhos K v1.0 and PROSITE searches are shown. DNAPK: DNA activated protein kinase, CDC2: Cell division cycle 2, RSK: 90 kDa ribosomal S6 kinase, TK: Tyrosine kinase, ATM: Ataxia Telangiectasia-Mutated.
(DOC)

Table S5 **List of primers used in this study.**
(PDF)

Figure S1 **Unrooted Maximum Likelihood phylogenetic tree of CK2β regulatory subunits.** The tree is based on the CLUSTAL alignment of 69 CK2β protein sequences. The clade clustering land plant CK2β is indicated. Non-land plant CK2β showing N-terminal extensions are in bold. Bootstrap values are displayed next to the corresponding nodes. The tree is drawn to scale, with branch lengths proportional to evolutionary distances. The scale bar indicates the estimated number of amino acid substitutions per site.
(PDF)

Figure S2 **Unrooted Neighbor Joining phylogenetic tree of CK2β regulatory subunits.** The tree is based on the CLUSTAL alignment of 69 CK2β protein sequences. The clade clustering land plant CK2β is indicated. Non-land plant CK2β showing N-terminal extensions are in bold. Bootstrap values are displayed next to the corresponding nodes. The tree is drawn to scale, with branch lengths proportional to evolutionary distances. The scale bar indicates the estimated number of amino acid substitutions per site.
(PDF)

Figure S3 **Quantification of Rab17 and β-casein phosphorylation with CK2α1/ΔNCK2β1 holoenzyme and increasing amounts of CK2β1 N-terminal domain (1–80).** Relative phosphorylation of Rab17 and β-casein with the holoenzyme composed by CK2α1/ΔNCK2β1 with increasing amounts of CK2β1 N-terminal domain (1–80) compared to phosphorylation of both substrates with CK2α1/ΔNCK2β1 holoenzyme alone (assigned a value of 1). The data plotted (mean ±SD) represent three independent experiments.
(PDF)

Figure S4 **Subcellular localization of CK2β1-GFP and ΔNCK2β1-GFP in** ***Agrobacterium*** **-infiltrated tobacco leaves and onion cells.** (A) Upper and middle panels show detail of fluorescent nucleus (60×) of cells from tobacco leaves infiltrated with a mixture of *Agrobacterium* suspensions harbouring the indicated constructs (CK2β1–GFP, ΔNCK2β1-GFP) and the gene silencing suppressor HcPro. In upper panel right, a confocal image of nuclear DAPI staining of cells transformed with CK2β1–GFP is shown (60×). General views (40×) of control cells infiltrated with GFP alone and HcPro are shown in the bottom of the panels. (B) Detail of fluorescent nucleus (60×) of onion cells transformed with CK2β1–GFP and ΔNCK2β1-GFP by particle bombardment. General views of onion cells (40×) transformed with GFP alone are shown on the right. In all cases epifluorescence and bright-field images (merged with epiflourescence) are shown.
(TIF)

Figure S5 **Immunodetection of CK2β1-GFP protein and ΔNCK2β1-GFP protein in transformed** ***N. benthamiana*** **leaves using anti-GFP antibody.** (A) Control and Cycloheximide treatment (CHX, 50 µM). Aliquots have taken at different times (30′, 1 h, 2 h and 4 h) (B) Control, Cycloheximide treatment (CHX, 50 µM) and proteasome inhibitor MG132 (100 µM). Aliquots have taken at different times (4 h and 8 h). In all analysis, 30 µg of total extracts has been loaded. The hybridation against Rubisco protein is shown as loading control.
(TIF)
