# Supplementary material for: Role of Plant-Specific N-Terminal Domain of Maize CK2β1 Subunit in CK2β Functions and Holoenzyme Regulation
Source: PLoS One. 2011 Jul 15;6(7):e21909. doi: 10.1371/journal.pone.0021909 (PMC3137599; doi:10.1371/journal.pone.0021909)
Supplement: Text S1 — Phylogenetic analysis of CK2β regulatory subunits. Phylogenetic analyses were performed on the basis of amino acid sequence alignments using two independent methods: Neighbor Joining (NJ) and Maximum Likelihood (ML). NJ analyses were implemented in MEGA 4.0 using the default settings [47] Prior to ML analysis; the best-fitting amino acid substitution model was selected using the Akaike information criterion as implemented in ProtTest v1.4 [26]. The resulting model: JTT with (i) an estimated proportion of invariable sites and (ii) a heterogeneous distribution of substitution rates across proteins with eight categories and an estimated shape parameter, was implemented in PHYML v3.0 to infer ML trees, using the subtree pruning and regrafting option to optimize tree topology searching [27]–[29]. To provide confidence on the resulting tree topology, a bootstrap analysis with 1,000 and 100 replicates in NJ and ML analyses, respectively, was performed. (DOC) [file pone.0021909.s001.doc]

**Supplementary Material and Methods**

**Phylogenetic analysis of CK2β regulatory subunits**

Phylogenetic analyses were performed on the basis of amino acid sequence alignments using two independent methods: Neighbor Joining (NJ) and Maximum Likelihood (ML). NJ analyses were implemented in MEGA 4.0 using the default settings [47] Prior to ML analysis; the best-fitting amino acid substitution model was selected using the Akaike information criterion as implemented in ProtTest v1.4 [26]. The resulting model: JTT with (i) an estimated proportion of invariable sites and (ii) a heterogeneous distribution of substitution rates across proteins with eight categories and an estimated shape parameter, was implemented in PHYML v3.0 to infer ML trees, using the subtree pruning and regrafting option to optimize tree topology searching [27-29]. To provide confidence on the resulting tree topology, a bootstrap analysis with 1,000 and 100 replicates in NJ and ML analyses, respectively, was performed.
