# Supplementary material for: Role of Plant-Specific N-Terminal Domain of Maize CK2β1 Subunit in CK2β Functions and Holoenzyme Regulation
Source: PLoS One. 2011 Jul 15;6(7):e21909. doi: 10.1371/journal.pone.0021909 (PMC3137599; doi:10.1371/journal.pone.0021909)
Supplement: Table S1 — Summary of genome databases searched for CK2β protein kinases. (DOC) [file pone.0021909.s002.doc]

**Table S1:** **Summary of genome databases searched for CK2β protein kinases.**
