# Supplementary material for: Role of Plant-Specific N-Terminal Domain of Maize CK2β1 Subunit in CK2β Functions and Holoenzyme Regulation
Source: PLoS One. 2011 Jul 15;6(7):e21909. doi: 10.1371/journal.pone.0021909 (PMC3137599; doi:10.1371/journal.pone.0021909)
Supplement: Table S2 — Summary of 34 land plant CK2β sequences. Sequence identifier refers to the UNIPROT database, excepting for species examined independently, in which case the accession from the corresponding database was indicated (Table S1). The * designs sequence incomplete at its N-terminal end. Some genes have been identified to encode for alternatively spliced variants. In such cases, only a single representative protein sequence is shown. (DOC) [file pone.0021909.s003.doc]

**Table S2**: **Summary of 34 land plant CK2β sequences.** Sequence identifier refers to the UNIPROT database, excepting for species examined independently, in which case the accession from the corresponding database was indicated (Table S1). The * designs sequence incomplete at its N-terminal end. Some genes have been identified to encode for alternatively spliced variants. In such cases, only a single representative protein sequence is shown.
