# Supplementary material for: Role of Plant-Specific N-Terminal Domain of Maize CK2β1 Subunit in CK2β Functions and Holoenzyme Regulation
Source: PLoS One. 2011 Jul 15;6(7):e21909. doi: 10.1371/journal.pone.0021909 (PMC3137599; doi:10.1371/journal.pone.0021909)
Supplement: Table S3 — Summary of 7 algae, 14 animal, 12 fungal and 2 protists CK2β sequences from representative species. Sequence identifier refers to the UNIPROT database, excepting for species examined independently, in which case the accession from the corresponding database was used (Table S1). The * indicates sequences incomplete at its N-terminal end. (DOC) [file pone.0021909.s004.doc]

**Table S3**: **Summary of 7 algae, 14 animal, 12 fungal and 2 protists CK2β sequences from representative species.** Sequence identifier refers to the UNIPROT database, excepting for species examined independently, in which case the accession from the corresponding database was used (Table S1). The * indicates sequences incomplete at its N-terminal end.
