# Supplementary material for: Role of Plant-Specific N-Terminal Domain of Maize CK2β1 Subunit in CK2β Functions and Holoenzyme Regulation
Source: PLoS One. 2011 Jul 15;6(7):e21909. doi: 10.1371/journal.pone.0021909 (PMC3137599; doi:10.1371/journal.pone.0021909)
Supplement: Table S4 — Summary of conserved motifs identified by MEME in plant CK2β subunits. Matches of motifs with specific kinase phosphorylation sites, predicted by NetPhos K v1.0 and PROSITE searches are shown. DNAPK: DNA activated protein kinase, CDC2: Cell division cycle 2, RSK: 90 kDa ribosomal S6 kinase, TK: Tyrosine kinase, ATM: Ataxia Telangiectasia-Mutated. (DOC) [file pone.0021909.s005.doc]

**Table S4**: **Summary of conserved motifs identified by MEME in plant CK2β subunits**. Matches of motifs with specific kinase phosphorylation sites, predicted by NetPhos K v1.0 and PROSITE searches are shown. DNAPK: DNA activated protein kinase, CDC2: Cell division cycle 2, RSK: 90 kDa ribosomal S6 kinase, TK: Tyrosine kinase, ATM: Ataxia Telangiectasia-Mutated.
