# Supplementary material for: Role of Plant-Specific N-Terminal Domain of Maize CK2β1 Subunit in CK2β Functions and Holoenzyme Regulation
Source: PLoS One. 2011 Jul 15;6(7):e21909. doi: 10.1371/journal.pone.0021909 (PMC3137599; doi:10.1371/journal.pone.0021909)
Supplement: Table S5 — List of primers used in this study. (PDF) [file pone.0021909.s006.pdf]

Table S5: **List of primers used in this study**

**Name**	**Sequence**
2H-Del1F	5’**GAATTC**TCTGATGGGGAAGATAC 3’
2H-Del1R	5’**GTCGAC**TCATGGCTTACGGATTTTC 3’
2H-Del2F	5’**GAATTC**TACAGGAACGTTGAGTT3’
2H-Del2R	5’**GTCGAC**TCATGGCTTACGGAT TTTC3’
2H-Del3F	5’**GAATTC**ATGCACCGAGACCGAGGCGTC3’
2H-Del3R	5’**GTCGAC**AACCACTCACAT CTGATTCTTCGC3’.
GFP-CK2B1_F	5’G**AGATCT**GATGCACCGAGACCGAGGCGTC3’
GFP-CK2B1_R	5’**ACTAGT**CATTGGCTTACG GATTTTGAACCCAAATACC3’
GFP-ΔNCK2B1_F	5’G**AGATCT**CTCTGATGGGG AAGATACTTCGTGG3’
GFP-ΔNCK2B1_R	5’C**AGATCT**ACCATTGGCTTACGGATTTTGAA CCCAAATACC3’
Myc-CK2B1_F	5’G**AGATCT**ATGCACCGAGACCGAGGCGTC3’
Myc-CK2B1_R	5’C**AGATCT**CATTGGCTTACGGATTTTGAACCCAAATACC3’
Entry-CK2α1_F	5’**GGGATCC**ATGTCCAAGGCCAGGGTCTACGC3
Entry-CK2α1_R	5’**GCTCGAG**TCACGCTCGCGTCCTGCTGTTC3’
