# Supplementary figures and images for: Role of Plant-Specific N-Terminal Domain of Maize CK2β1 Subunit in CK2β Functions and Holoenzyme Regulation
Source: PLoS One. 2011 Jul 15;6(7):e21909. doi: 10.1371/journal.pone.0021909 (PMC3137599; doi:10.1371/journal.pone.0021909)

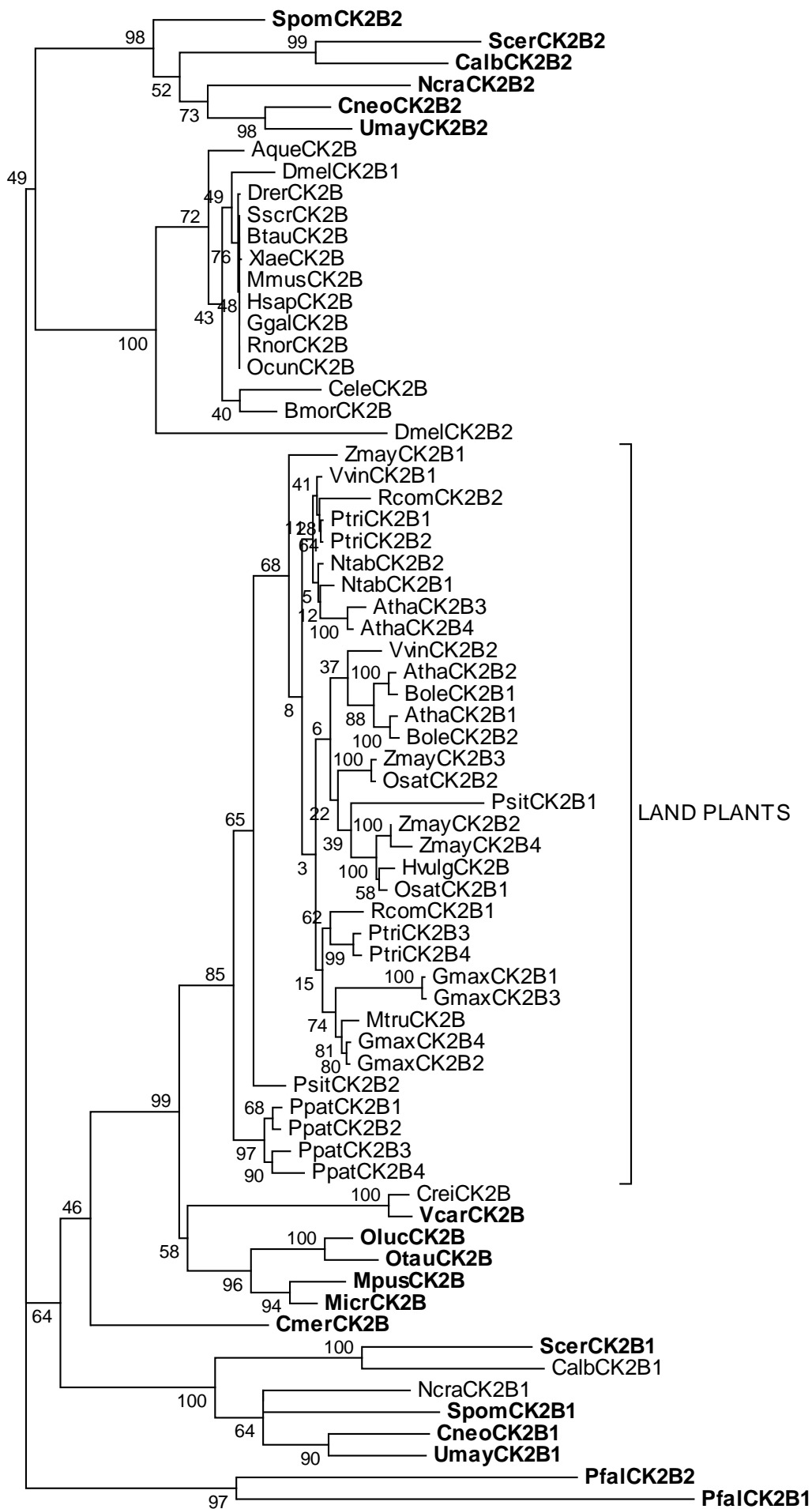

Supplement: Figure S1 — Unrooted Maximum Likelihood phylogenetic tree of CK2β regulatory subunits. The tree is based on the CLUSTAL alignment of 69 CK2β protein sequences. The clade clustering land plant CK2β is indicated. Non-land plant CK2β showing N-terminal extensions are in bold. Bootstrap values are displayed next to the corresponding nodes. The tree is drawn to scale, with branch lengths proportional to evolutionary distances. The scale bar indicates the estimated number of amino acid substitutions per site. (PDF) [file pone.0021909.s007.pdf]

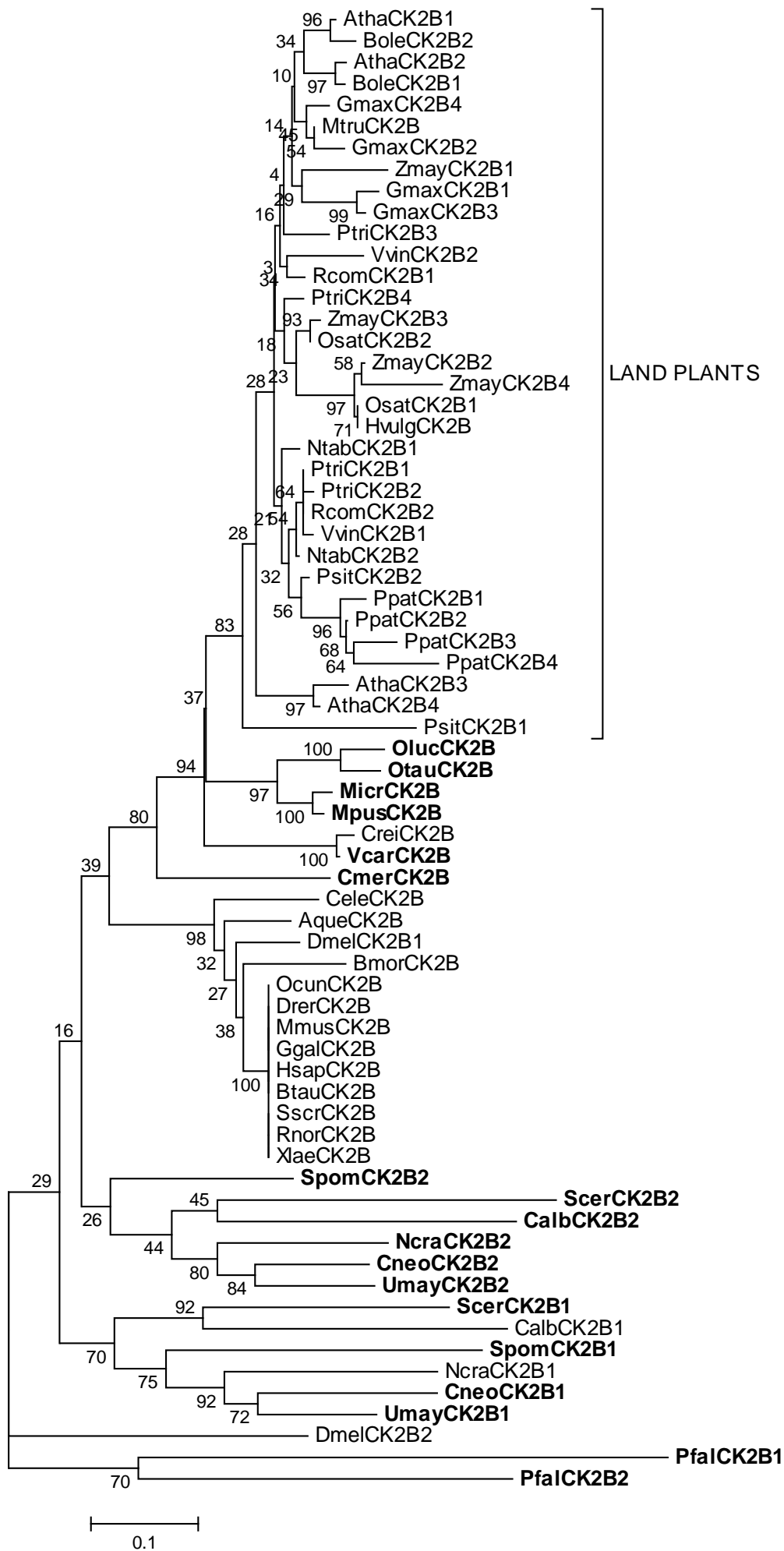

Supplement: Figure S2 — Unrooted Neighbor Joining phylogenetic tree of CK2β regulatory subunits. The tree is based on the CLUSTAL alignment of 69 CK2β protein sequences. The clade clustering land plant CK2β is indicated. Non-land plant CK2β showing N-terminal extensions are in bold. Bootstrap values are displayed next to the corresponding nodes. The tree is drawn to scale, with branch lengths proportional to evolutionary distances. The scale bar indicates the estimated number of amino acid substitutions per site. (PDF) [file pone.0021909.s008.pdf]

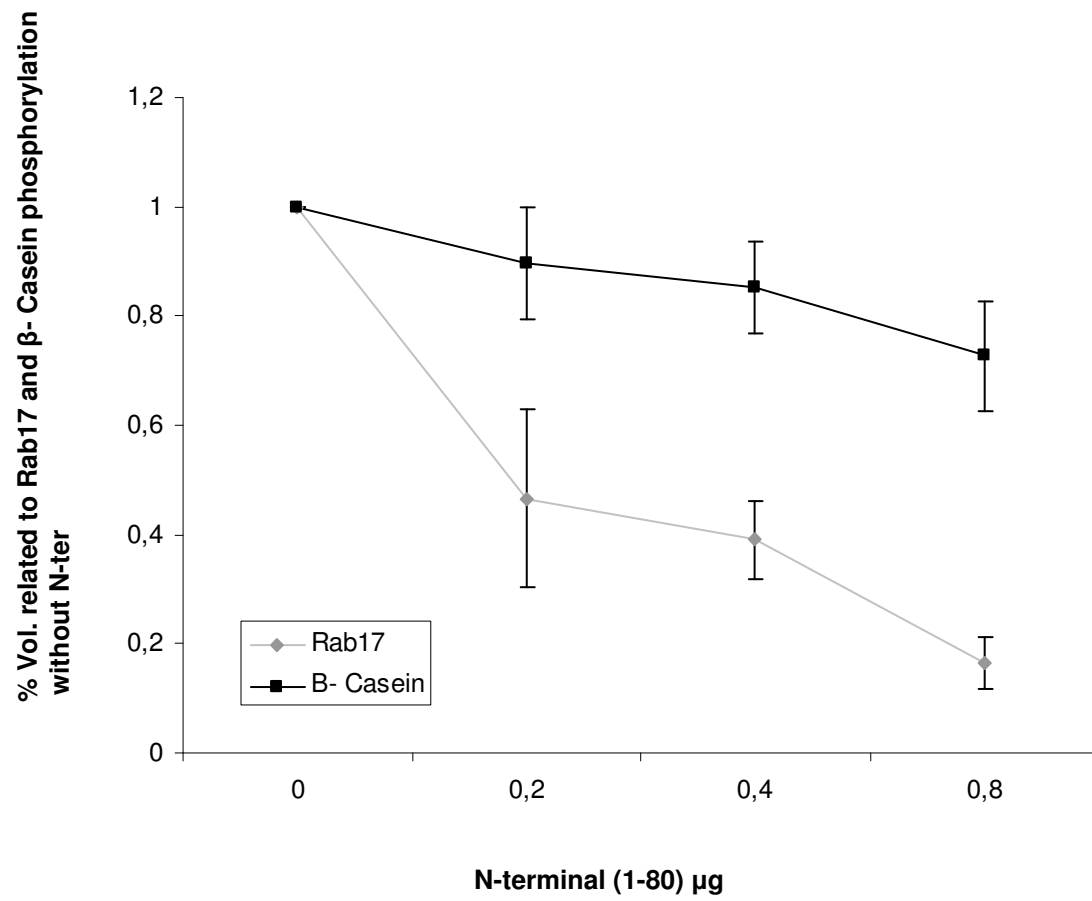

Supplement: Figure S3 — Quantification of Rab17 and β-casein phosphorylation with CK2α1/ΔNCK2β1 holoenzyme and increasing amounts of CK2β1 N-terminal domain (1–80). Relative phosphorylation of Rab17 and β-casein with the holoenzyme composed by CK2α1/ΔNCK2β1 with increasing amounts of CK2β1 N-terminal domain (1–80) compared to phosphorylation of both substrates with CK2α1/ΔNCK2β1 holoenzyme alone (assigned a value of 1). The data plotted (mean ±SD) represent three independent experiments. (PDF) [file pone.0021909.s009.pdf]

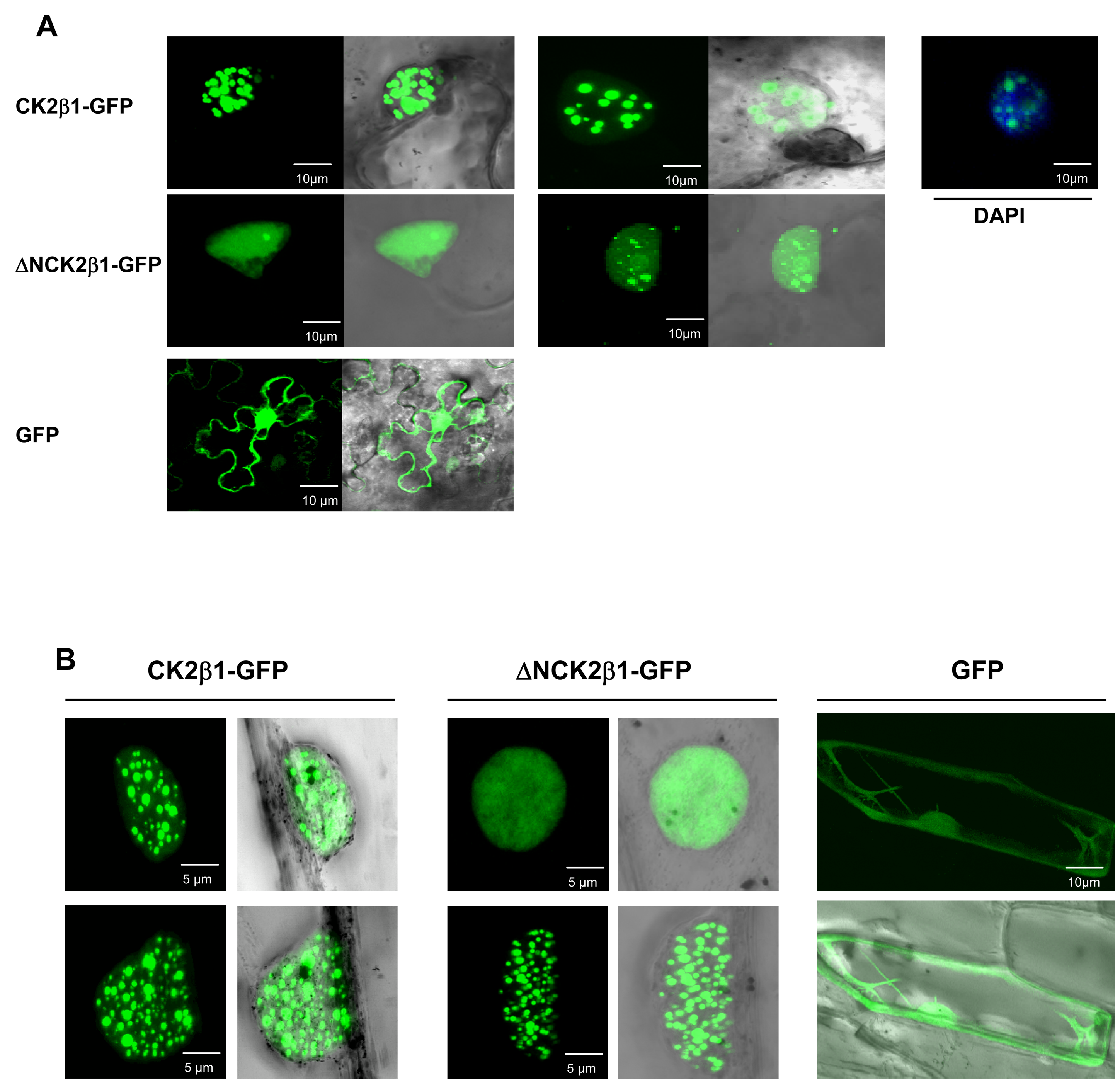

Supplement: Figure S4 — Subcellular localization of CK2β1-GFP and ΔNCK2β1-GFP in Agrobacterium -infiltrated tobacco leaves and onion cells. (A) Upper and middle panels show detail of fluorescent nucleus (60×) of cells from tobacco leaves infiltrated with a mixture of Agrobacterium suspensions harbouring the indicated constructs (CK2β1–GFP, ΔNCK2β1-GFP) and the gene silencing suppressor HcPro. In upper panel right, a confocal image of nuclear DAPI staining of cells transformed with CK2β1–GFP is shown (60×). General views (40×) of control cells infiltrated with GFP alone and HcPro are shown in the bottom of the panels. (B) Detail of fluorescent nucleus (60×) of onion cells transformed with CK2β1–GFP and ΔNCK2β1-GFP by particle bombardment. General views of onion cells (40×) transformed with GFP alone are shown on the right. In all cases epifluorescence and bright-field images (merged with epiflourescence) are shown. (TIF) [file pone.0021909.s010.tif]

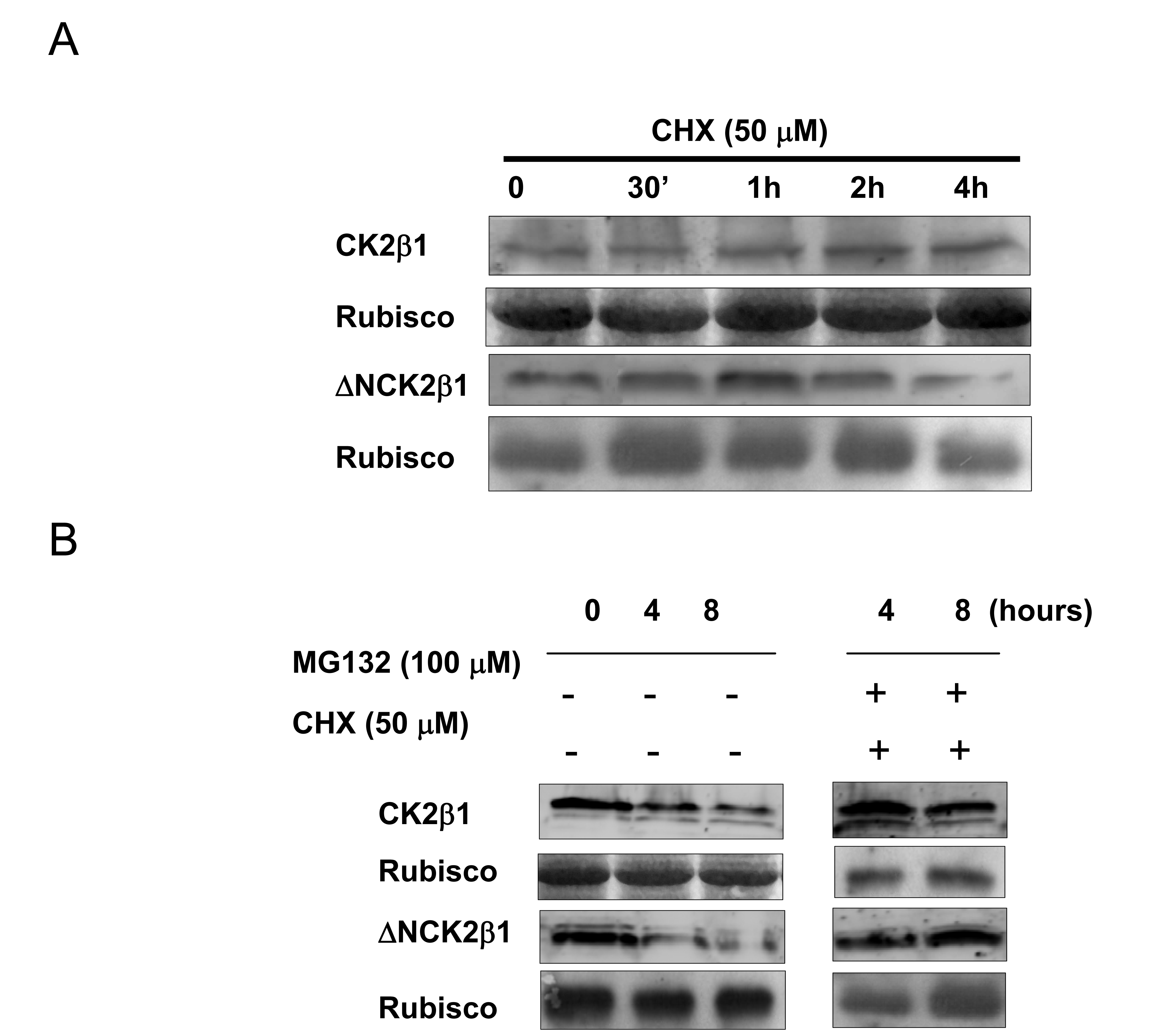

Supplement: Figure S5 — Immunodetection of CK2β1-GFP protein and ΔNCK2β1-GFP protein in transformed N. benthamiana leaves using anti-GFP antibody. (A) Control and Cycloheximide treatment (CHX, 50 µM). Aliquots have taken at different times (30′, 1 h, 2 h and 4 h) (B) Control, Cycloheximide treatment (CHX, 50 µM) and proteasome inhibitor MG132 (100 µM). Aliquots have taken at different times (4 h and 8 h). In all analysis, 30 µg of total extracts has been loaded. The hybridation against Rubisco protein is shown as loading control. (TIF) [file pone.0021909.s011.tif]
